# Recent advances in understanding the role of FOXO3

**DOI:** 10.12688/f1000research.15258.1

**Published:** 2018-08-31

**Authors:** Renae J. Stefanetti, Sarah Voisin, Aaron Russell, Séverine Lamon

**Affiliations:** 1 Wellcome Centre for Mitochondrial Research, Newcastle University, Newcastle upon Tyne, UK; 2 Institute for Health and Sport, Victoria University, Footscray, Australia; 3 Institute for Physical Activity and Nutrition, School of Exercise and Nutrition Sciences, Deakin University, Geelong, Australia

**Keywords:** FOXO3, transcription factor, skeletal muscle, protein turnover, longevity, microRNA

## Abstract

The forkhead box O3 (FOXO3, or FKHRL1) protein is a member of the FOXO subclass of transcription factors. FOXO proteins were originally identified as regulators of insulin-related genes; however, they are now established regulators of genes involved in vital biological processes, including substrate metabolism, protein turnover, cell survival, and cell death. *FOXO3* is one of the rare genes that have been consistently linked to longevity in *in vivo* models. This review provides an update of the most recent research pertaining to the role of FOXO3 in (i) the regulation of protein turnover in skeletal muscle, the largest protein pool of the body, and (ii) the genetic basis of longevity. Finally, it examines (iii) the role of microRNAs in the regulation of FOXO3 and its impact on the regulation of the cell cycle.

## Introduction

The forkhead box O3 (FOXO3, or FKHRL1) protein is one of about 40 forkhead box (FOX) transcription factors encoded by the mammalian genome ^1^. FOX transcription factors are versatile proteins containing an evolutionarily conserved winged helix DNA-binding motif of about 100 residues at the N-terminal region, the forkhead (FKH) domain ^2– 4^. FOXO3 belongs to the FOXO subclass (made of FOXO1, FOXO3, FOXO4, and FOXO6), which historically is known to regulate insulin signaling (comprehensively reviewed in 5, 6). Numerous regulatory processes, including phosphorylation, acetylation, ubiquitination, methylation ^7, 8^, and microRNA (miRNA) binding ^9^, can modulate FOXO3 transcriptional activity. Of particular interest for human health, FOXO3 is under the control of the phosphatidylinositol 3-kinase (PI3K)/Akt signaling pathway ^10^. In its non-phosphorylated form, FOXO3 localizes at the nucleus where it regulates gene transcription. Activation of the PI3K/Akt pathway results in the phosphorylation of FOXO3 at three conserved residues ^11^. This usually causes its exclusion from the nucleus and stops the transcriptional activation of its target genes ^1, 12^. Phosphorylated FOXO3 therefore represents the inactive form of the protein. Through PI3K/Akt, FOXO3 mediates biological processes that are essential for health over the lifespan, including substrate metabolism, protein turnover, cell survival, and cell death ^11, 13, 14^.

Our research group investigates skeletal muscle wasting and miRNA-mediated regulation. Although the involvement of FOXO3 in these processes is undeniable, these two topics have not been reviewed in the specific context of FOXO3. In addition, the role and regulation of FOXO3 in the genetics of longevity constitute a very dynamic research field, justifying the need for an updated review encompassing the research articles published over the last 3 years.

## FOXO3 and the regulation of skeletal muscle homeostasis

FOXO proteins are expressed across multiple tissues of the body but their expression level, function, and targets are tissue specific. In mice, *Foxo3* mRNA is enriched in the heart, brain, spleen, and kidney and, to a certain extent, skeletal muscle ^15^. FOXO3 is a key player in the control of skeletal muscle protein turnover and a central effector of PI3K/Akt signaling, the main regulator of protein synthesis and degradation in the muscle ^16^. In anabolic conditions, Akt phosphorylates FOXO3 and suppresses its transcriptional activity. FOXO3 inhibition in turn reduces the expression of the muscle-enriched members of the ubiquitin-proteasome system, atrogin-1 (FBXO32) and muscle RING finger 1 (MURF1) ^17^, which promote muscle protein degradation. In addition, upon Akt activation, FOXO proteins may play a role in a negative feedback loop that inhibits Akt to maintain the cell homeostatic balance. In non-mammalian cells, FoxO orthologues inhibit the activity of the mechanistic target of rapamycin complex 1 (mTORC1) ^18, 19^, which drives muscle protein synthesis downstream of Akt ^16^. In mammalian tissue, FOXO proteins reduce mTORC1 activity, thereby activating Akt ^20^. FOXO proteins therefore may play an intricate role in balancing Akt and mTORC1 activities in response to changing metabolic conditions.

In mouse ^21– 23^ and human ^24^ skeletal muscle, FOXO3 mRNA or total protein expression or both are upregulated under artificially induced catabolic conditions such as limb suspension or calorie restriction, suggesting that FOXO3 contributes to muscle wasting in these models. Recent rodent studies using immobilization models point toward myofiber type-specific regulation of FOXO3 ^23, 25^. However, a recent study showed no difference in *FOXO3* mRNA levels or in the cytoplasmic levels of the inactive phosphorylated FOXO3 protein in overweight young men subjected to energy restriction ^26^, potentially because other factors pertaining to insulin signaling may be at play. The complexity of FOXO protein regulation and the redundancy of FOXO alleles suggest that changes in gene and protein expression levels need to be interpreted with care, as they may not provide direct insights into the mechanistic processes at play.

Disease-induced catabolic states are also characterized by increased FOXO3 expression levels. *Foxo3* mRNA levels were elevated in the late symptomatic stage of two mouse models of spinal muscular atrophy ^27^. FOXO3 was also identified in a network-based analysis comparing serum proteomics in patients with Duchenne muscular dystrophy and age-matched controls ^28^, suggesting potential for FOXO3 as a protein biomarker to monitor disease progression in conditions with severe skeletal muscle atrophy. Patients with chronic obstructive pulmonary disease displayed an increased ratio of phosphorylated FOXO3 to total FOXO3 in their muscle when compared with healthy controls with or without sarcopenia ^29^.

Whereas higher levels of FOXO3 are typically observed in pathological catabolic conditions, FOXO3 expression patterns are not upregulated in healthy old muscle. Sarcopenic mice display no change in nuclear or total FOXO3 protein expression despite reduced phosphorylation levels that might be indicative of higher FOXO3 activity ^30^. We and others showed that *FOXO3* mRNA ^31, 32^ and FOXO3 nuclear protein levels decreased in old human skeletal muscle ^33^ whereas total or phosphorylated FOXO3 protein expression did not change ^34^. It is generally accepted that sarcopenia cannot be attributed to an upregulation of the proteolytic system or an induction of FOXO3 ^35^. Therefore, in aging muscle, FOXO3 may be similarly or even less active than in younger muscle or in models of artificially or disease-induced atrophy. Overall, these results confirm the idea that a series of upstream regulatory factors that inhibit FOXO3 transcriptional activity, including peroxisome proliferator-activated receptor gamma coactivator-1-alpha (PGC-1α) and PI3K/Akt itself ^20^, protect the muscle from aging-related atrophy ^36, 37^. In addition, the role of FOXO3 in the process of muscle aging might rely on a fine balance between the regulation of protein turnover ^20^ and other, protective anti-aging processes, such as the maintenance of the pool of skeletal muscle stem cells ^38^, which is discussed below.

## FOXO3 and the genetics of longevity

*FOXO3* is among the few genes associated with human longevity that have been consistently replicated. Genetic variants of *FoxO3* are associated with exceptional longevity in worms, flies, and mammals ^39^. In humans, *FOXO3* hosts about 40 common, non-coding single-nucleotide polymorphisms (SNPs) that have been consistently associated with longevity in Caucasian ^40– 44^ and Asian ^43, 45, 46^ populations. FOXO3 gene and protein expression is associated with age-related phenotypes in multiple tissues ^47^. For example, an age-dependent decrease in FOXO3 protein contributes to the loss of anti-inflammatory behavior in macrophages of old mice ^48^. Additionally, FOXO3-deficient mice demonstrate signs of pronounced neural activation, apoptosis, and enteric neuronal loss, indicative of premature aging of the enteric nervous system ^48^. FOXO3 overexpression also facilitates autophagy, a process of degradation and recycling of cytoplasmic proteins and organelles that is essential for healthy aging in multiple tissues, including skeletal muscle ^49^. However, the key role of FOXO3 in aging seems to be via the maintenance of stem cell homeostasis ^50^, including in the brain ^51^, blood ^52^, and skeletal muscle ^38^. Whether the modulation of molecular pathways involved in the age-dependent deterioration of stem cell function can reverse aging phenotypes remains controversial ^53^. In skeletal muscle stem cells, termed “satellite cells”, FOXO3 enhances stem cell self-renewal via the activation of Notch signaling, maintaining an available pool of satellite cells that have divided but retain their undifferentiated state ^38^. In fact, FOXO proteins play a dual role, and in situations of cellular damage, they can induce cell cycle arrest and senescence while independently repressing stemness signaling ^54^. Yet, in humans, despite consistent associations between *FOXO3* genetic variants and exceptional longevity ^40– 46^, a possible link between *FOXO3* and healthy aging remains unclear. For example, the G allele of a longevity variant of *FOXO3* was associated with a 10% reduction in all-cause mortality in a prospective cohort study of 3,584 older American men ^55^. Moreover, in a cross-sectional study including more than 30,000 individuals, the G allele of another longevity variant of *FOXO3* was associated with a decrease in concentration of circulating insulin-like growth factor-1 (IGF-1), a marker of insulin resistance and chronic disease ^56^. Smaller-scale studies have yielded mixed results, albeit showing consistent trends. In the seminal study on *FOXO3* and longevity (n = 615), carriers of *FOXO3* longevity variants had lower prevalence of coronary heart disease and insulin resistance ^43^, echoing similar findings on hypertension in Japanese-American women ^57^. In two recent studies on older Swedes (n = 1,520) ^58^ and Danes (n = 1,088) ^59^, carriers of the longevity alleles had better self-rated health even after accounting for cardiovascular disease incidence ^58^, higher activity of daily living, and fewer bone fractures ^59^. However, these latter findings did not survive adjustment for multiple testing ^59^. Similarly, two functional longevity variants of *FOXO3* failed to associate with mortality and age-related phenotypes in another sample of 643 long-lived Danes ^60^. A recent whole-genome sequencing study also found no differences in genotype distribution at *FOXO3* longevity variants between 511 healthy elderly and 686 controls ^61^. However, these small sample sizes suggest that these negative findings may partly reflect a lack of statistical power.

Two recent studies provide insight into how the longevity variants of *FOXO3* may act at the molecular and cellular levels. In carriers of the G allele of a longevity variant of *FOXO3*, the *FOXO3* gene was physically closer to its neighboring genes, and when exposed to stress, *FOXO3* mRNA expression in lymphoblastoid cell lines derived from carriers increased more than in cell lines derived from non-carriers ^62^. In line with those findings, another study showed that the same genetic variant has enhancer functions and that the G allele allows the creation of a novel transcription factor binding site, which induces *FOXO3* mRNA expression in response to diverse stress stimuli ^63^.

Collectively, these results suggest that FOXO3 genetic variants contribute to reaching old age, but there is a paucity of human studies that are sufficiently powered to demonstrate the role of FOXO3 in healthy aging ^60^. One mechanism of action of the *FOXO3* SNPs was only recently uncovered and involves a complex “interactome” whereby cellular stress causes *FOXO3* to move close and physically interacts with no fewer than 46 flanking genes on chromosome 6 ^64^. Rather than just *FOXO3*, the strong association of *FOXO3* with longevity might rely on the central position of *FOXO3* in a chromatin domain containing essential genes involved in cell resilience, including autophagy, stress response, energy/nutrient sensing, cell proliferation, apoptosis, and stem cell maintenance ^62– 64^.

## MicroRNA-mediated regulation of FoxO3

MiRNAs are regulatory, small non-coding RNAs. The physiological effect of most miRNAs is based on the post-transcriptional regulation of mRNA expression or the inhibition of protein translation ^65^. Whereas correlations are often made between the expression levels of a specific miRNA and its predicted gene and protein targets, miRNA/mRNA direct regulatory relationships can be confirmed only via the means of luciferase reporter experiments *in vitro*. All of the miRNA/ *FOXO3* regulatory relationships discussed below were confirmed by luciferase validation.

### MicroRNA regulation in autophagy and apoptosis

Increasing exogenous levels of miR-182 decreased FOXO3 mRNA and protein expression in C _2_C _12_ myotubes ^66^ and FOXO3 protein levels in hair cells ^67^. Downstream responses included an attenuation of the mRNA levels of FOXO3 catabolic targets *Fbxo32*, autophagy-related protein 12 ( *Atg12*), Cathepsin L ** ( *Ctsl*), and microtubule-associated protein light chain 3 ( *Lc3*) following atrophy-inducing dexamethasone treatment in C _2_C _12_ myotubes ^66^ as well as an attenuation of cisplatin-induced apoptosis and increase in cell survival in hair cells ^67^. Similar to miR-182, elevated levels of miR-34a reduced FOXO3 protein levels and attenuated lipopolysaccharide-induced autophagic activity in alveolar epithelial type **II (**AT-II) cells ^68^. The opposite effects were observed when miR-34a levels were reduced. Other miRNA targets mediating apoptosis via FOXO3 include miR-223 and miR-155 ^69– 71^. Apoptosis of peripheral blood macrophages is decreased in patients with tuberculosis, while isolated human macrophages transfected with mycobacterium tuberculosis (Mtb) strain H37Rv displayed an increase in endogenous miR-223 levels. These results suggest an association between elevated levels of miR-223 and reduced apoptosis. In support of this, the overexpression of miR-223 in isolated human macrophages reduced apoptosis and suppressed FOXO3 protein levels. The miR-223 inhibitory effect on apoptosis was counteracted by FOXO3 overexpression ^71^. Finally, expression levels of miR-155 are increased in renal tissues of rats that have undergone ischemia/reperfusion injury as well as in hypoxia/reoxygenation injury-induced human kidney proximal tubules epithelial (HK2) cells ^70^. Overexpressing miR-155 in HK2 cells repressed FOXO3 mRNA and protein levels, increased caspase-1, interleukin-1 beta (IL-1β), and IL-18 mRNA and protein levels, and increased pyroptosis, a response that was attenuated by the suppression of miR-155 ^70^.

### MicroRNA regulation in cell proliferation and growth

Prostate cancer (PC) tissue and primary prostate epithelial cell lines (PC cells) display increased expression levels of endogenous miR-592 ^72^ and miR-1307 ^73^. Overexpression of these two miRNAs in PC cells inhibited FOXO3 protein levels and increased cell proliferation whereas suppressing their expression reversed these effects. Similarly, miR-592 levels were elevated, and FOXO3 mRNA and protein reduced, in colorectal cancer (CRC) tissues and cells ^74^. In contrast, lentiviral-induced inhibition of miR-592 attenuated CRC cell proliferation and clonogenicity ^74^. Overexpressing miR-551b, an miRNA that has elevated levels in ovarian cancer tissue, in isolated primary ovarian cancer (OVCa) cells increased proliferation, invasion, and chemoresistance of OVCa stem cells via the suppression of FOXO3 and TRIM31 proteins ^75^. *In vivo*, miR-551b inhibition increased the susceptibility of OVCa cells to the chemotherapy drug cisplatin and prolonged the survival of host mice ^75^. In contrast, miR-498 levels were decreased in ovarian cancer tissue. Overexpressing miR-498 attenuated OVCa cell proliferation and was associated with a decrease in *Cyclin D1* and increase in *p27* expression, indicating that more cells remained in the G _0_/G _1_ phases of the cell cycle ^76^. Of particular interest was the observation that the binding of miR-498 to *FOXO3* 3′-untranslated region increased its expression levels, an effect that is rare but not without precedent ^77, 78^. Finally, overexpression of miR-142-5p in chicken primary myoblasts ^79^ and miR-155-5p in human foreskin fibroblasts ^80^ increased cell proliferation. This effect was mediated via a decrease in FOXO3 in both cell types. In the myoblasts, overexpressing miR-142-5p was associated with an increase in genes known to regulate growth such as *IGF1R*, *IGF2R*, *IGF2BP2*, *MTH10*, and *PGK1*. In the fibroblasts, overexpressing miR-155-5p inhibited cyclin-dependent kinase inhibitor 1B (CDKN1B). These effects were reversed by the inhibition of endogenous levels of these two miRNAs.

This series of recent studies confirms that numerous miRNAs regulate FOXO3, often in a tissue-, cell-, or disease-specific manner. However, to date, the direct miRNA/FOXO3 relationships have been assessed only under non-physiological and *in vitro* conditions. Although this fundamental work is essential and has significantly increased our understanding of the post-transcriptional regulation of FOXO3, research should now shift toward the *in vivo* regulation of FOXO3 targeting miRNAs in suitable animal models of human disease. Understanding how miRNAs regulate FOXO3 activity is of interest for many fields of biomedical research, as miRNAs potentially constitute novel and effective targets for human therapy ^81^.

### Mechanism protecting FOXO3 from microRNA regulation

Two mechanisms have been identified that protect FOXO3 from being targeted by certain miRNAs. The Foxo3 pseudogene (Foxo3P) and the Foxo3 circular RNA (circ-Foxo3) act as a “sponge” to bind miRNAs that normally would target FOXO3. Several miRNAs, including miR-22, miR-136, miR-138, miR-149, miR-433, miR-762, miR-3614-5p, and miR3622b-5p, all interact with FOXO3 ^82^ but do not cause transcript degradation. Competition assays and luciferase reporter assays revealed that Foxo3P and circ-Foxo3 can compete with Foxo3 for binding to these miRNAs. This competitive inhibition results in an increase in FOXO3 translation. Foxo3P and circ-Foxo3 are endogenously expressed in non-cancerous lines such as BEAS2B, HaCaT, and MCF-10A. When these cells are transfected with Foxo3, Foxo3P, or circ-Foxo3 and exposed to hydrogen peroxide (H _2_O _2_), cell survival decreases. Additionally, nude mice injected with MDA-MB-231 cells overexpressing Foxo3, Foxo3P, or circ-Foxo3 have small tumor growth, demonstrating that Foxo3P or circ-Foxo3 has functional consequences similar to those of Foxo3.

## Conclusions

FOXO3 has versatile functions in human health and disease, and recent research offers new insights into the molecular mechanisms underlying the role and regulation of this essential transcription factor. Over the last 3 years, an important part of FOXO3 research has focused on longevity studies combining population epidemiology and molecular investigations, and the aim has been to pinpoint the mechanisms that underlie the role of FOXO3 in longevity. Simultaneously, numerous new findings highlight the important role of miRNAs in the regulation of FOXO3 and their implication in the regulation of cell cycle-related processes. Overall, despite a strong association of FOXO3 with aging phenotypes, its role in healthy aging remains unclear, especially in skeletal muscle. This may constitute an exciting focus for research in the future.
